# Poly(ADP-ribose) polymerase as a novel regulator of 17β-estradiol-induced cell growth through a control of the estrogen receptor/IGF-1 receptor/PDZK1 axis

**DOI:** 10.1186/s12967-015-0589-7

**Published:** 2015-07-17

**Authors:** Hogyoung Kim, Abdelmetalab Tarhuni, Zakaria Y Abd Elmageed, A Hamid Boulares

**Affiliations:** The Stanley Scott Cancer Center, Louisiana State University Health Sciences Center, 1700 Tulane Ave, New Orleans, LA 70112 USA; Tulane University Cancer Center, New Orleans, LA 70112 USA

**Keywords:** PARP, 17β-estradiol, Gene regulation, Cell growth, Breast cancer, PDZK1, IGF-1R, MCF-7 cells

## Abstract

**Background:**

We and others have extensively investigated the role of PARP-1 in cell growth and demise in response to pathophysiological cues. Most of the clinical trials on PARP inhibitors are targeting primarily estrogen receptor (ER) negative cancers with BRCA-deficiency. It is surprising that the role of the enzyme has yet to be investigated in ER-mediated cell growth. It is noteworthy that ER is expressed in the majority of breast cancers. We recently showed that the scaffolding protein PDZK1 is critical for 17β-estradiol (E_2_)-induced growth of breast cancer cells. We demonstrated that E_2_-induced PDZK1 expression is indirectly regulated by ER and requires IGF-1 receptor (IGF-1R).

**Methods:**

The breast cancer cell lines MCF-7 and BT474 were used as ER(+) cell culture models. Thieno[2,3-c]isoquinolin-5-one (TIQ-A) and olaparib (AZD2281) were used as potent inhibitors of PARP. PARP-1 knockdown by shRNA was used to show specificity of the effects to PARP-1.

**Results:**

In this study, we aimed to determine the effect of PARP inhibition on estrogen-induced growth of breast cancer cells and examine whether the potential effect is linked to PDZK1 and IGF-1R expression. Our results show that PARP inhibition pharmacologically by TIQ-A or olaparib or by PARP-1 knockdown blocked E_2_-dependent growth of MCF-7 cells. Such inhibitory effect was also observed in olaparib-treated BT474 cells. The effect of PARP inhibition on cell growth coincided with an efficient reduction in E_2_-induced PDZK1 expression. This effect was accompanied by a similar decrease in the cell cycle protein cyclin D1. PARP appeared to regulate E_2_-induced PDZK1 at the mRNA level. Such regulation may be linked to a modulation of IGF-1R as PARP inhibition pharmacologically or by PARP-1 knockdown efficiently reduced E_2_-induced expression of the receptor at the protein and mRNA levels.

**Conclusions:**

Overall, our results show for the first time that PARP regulates E_2_-mediated cell growth by controlling the ER/IGF-1R/PDZK1 axis. These findings suggest that the relationship between ER, PDZK1, and IGF-1R may be perturbed by blocking PARP function and that PARP inhibitors may be considered in clinical trials on ER(+) cancers.

## Background

It is well established that estrogen receptor (ER) is expressed in the majority of breast cancers and are responsive to standard therapy with tamoxifen as the leading drug [1]. Despite the success of this therapy, reduction of breast cancer recurrence is only by 50% and an even lower reduction in mortality rate (~30%) [1, 2]. Accordingly, finding new therapeutic targets is urgently needed. Achieving this goal requires the identification of new players in the complex process of the disease. We have recently shown that PDZK1, a scaffold protein, plays an important role in estrogen-induced growth of breast cancer cells and demonstrated a strong correlation between the expression of the protein and human breast malignancy [3]. We also reported that *PDZK1* gene expression is not a direct product of ER stimulation; rather, it requires the expression and function of IGF-1 receptor (IGF-1R) [3]. PDZK1 appears to harbor oncogenic activity and promote cell growth by enhancing EGFR-stimulated MEK/ERK1/2 signaling and IGF-induced Akt phosphorylation [4]. Interestingly, PDZK1 plays this important role through stabilization of the integrity of Akt, Her2/Neu, and EGFR [4]. The co-chaperone Cdc37 appears to play an important role in PDZK1-mediated stability of Akt [4]. These aforementioned findings demonstrated a novel relationship between PDZK1, Akt, Her2/Neu, EGFR and Cdc37 in breast cancer unraveling a new axis that can be targeted therapeutically to reduce the burden of human breast cancer.

Poly (ADP-ribose) polymerase (PARP)-1, a member of the PARP family of proteins, has initially been described as a DNA repair enzyme playing primarily as a regulatory protein controlling traffic of DNA repair proteins during base excision repair [5, 6]. A prominent function of this enzyme is in cell death both as an effector and as a substrate to some of the caspases [7]. We demonstrated many years ago that cleavage of PARP-1 is critical for the normal progression of the apoptotic process and that interference with such cleavage enhances cell death and may even cause a switch to necrosis [7, 8]. Increasing evidence from our laboratory and many others demonstrate an important role for this enzyme in tissue injury associated with oxidative stress and inflammation including asthma and atherosclerosis [8–13]. PARP-1 is thought to participate in inflammation by regulating the expression of several inflammatory factors including adhesion molecules, TNF-α, interleukins, and inducible nitric oxide synthase (iNOS) most of which are controlled by NF-κB (4). PARP inhibitors have shown great potential against breast and ovarian cancers especially those with BRCA mutations [14]. The combination of PARP inhibitors with DNA damaging chemotherapeutic drugs have shown to induce the specific demise of BRCA-deficient cancer cells leading to a synthetic lethality phenotype while sparing the life of normal cells [15]. Many clinical trials have demonstrated efficacy of PARP inhibitors and their potential as therapeutic strategy that can be utilized in the clinic [14, 15]. However, the focus on BRCA-deficient breast cancer prevented the examination of the effects of PARP inhibitors on ER positive breast cancer cells and, as a result, may be reducing the full therapeutic potential of these drugs.

In the present study we wished to determine the effect of PARP inhibition pharmacologically or PARP-1 knockdown on estrogen-induced growth of the ER positive breast cancer cell line MCF-7 and BT474 and to examine whether the potential effect was related to a modulation of E_2_-induced PDZK1 expression.

## Methods

### Materials

DMEM, penicillin, streptomycin, and fetal bovine serum (FBS) were purchased from Invitrogen (Camarillo, CA, USA). Charcoal/dextran-treated FBS (CDSS) was purchased from Hyclone (Logan, Utah, USA); 17β-Estradiol (E_2_), and the PARP-1 inhibitor TIQ-A were from Sigma-Aldrich (St. Louis, MO, USA); olaparib (AZD2281) was from Selleckchem (Pittsburgh, PA, USA); IGF-1R inhibitor AG1024 was from Calbiochem (San Diego, CA, USA); PARP-1 and PDZK-1 shRNA expressing lentiviral vectors and the control virus were from Santa Cruz Biotechnology (Santa Cruz, CA, USA). Unless otherwise indicated, all other drugs were purchased from Sigma-Aldrich.

### Cell culture, cell proliferation, cell survival, transfection, immunoblot analysis, and RT-PCR

The ER positive breast Cancer cell line MCF-7 was obtained from ATCC (Manassas, VA, USA). The second ER positive breast cancer cell line BT474 was originally obtained from ATCC but provided by Dr. Wanguo Liu (LSUHSC). The two cell lines were cultured according to ATCC instructions. These cell lines are authenticated by ATCC using short tandem repeat (STR) profiling. This PCR-based approach permits the authentication of human cell lines with high resolution down to the individual donor. Upon receipt from ATCC, the morphology was confirmed by microscopy and population-doubling times were determined using the trypan blue dye exclusion method. After cells reached 70% confluence, complete DMEM medium was changed to DMEM supplemented with 5% CDSS followed by E_2_ (1 nM) treatment for the indicated time periods. Cells were also treated with TIQ-A, olaparib, or AG1024. Cell proliferation was measured by MTT assay after 2 days of treatment as previously described [3]. In some experiments, cells were transduced with a lentiviral vector encoding control shRNA or shRNA targeting human PARP-1 or PDZK1 (Santa Cruz Biotechnology) according to the manufacturer’s instructions. Cells were selected with puromycin dihydrochloride (Santa Cruz Biotechnology) and treated as described above. After the treatments, cells were collected and subjected to total RNA or protein preparation. Isolated RNA was reverse-transcribed and the resulting cDNA was subjected to conventional or quantitative PCR with primer sets purchased from IDT (San Jose, CA, USA) specific to human PDZK1, IGF-1R and β-actin as described [3]. Protein extracts were subjected to immunoblot analysis with antibodies to PDZK1 (EPR3751 clone) (Novus Biological), PARP-1 (BD Biosciences, San Jose, CA), cyclin D (Santa Cruz Biotechnology) or GADPH (Santa Cruz Biotechnology). The immunoblots were incubated with the appropriate secondary antibodies and signals were detected by ECL (Pierce, Rockford, IL, USA).

### Statistical analysis

Data are presented as mean ± SEM from at least three separate experiments. Comparisons between multiple groups were performed with one-way ANOVA with Bonferroni’s test using GraphPad software, Version 5 (La Jolla, CA, USA). Statistical significance was considered at *p* < 0.05.

## Results and discussion

### PARP inhibition blocks E_2_-dependent growth of MCF-7 cells

**Figure 1:**
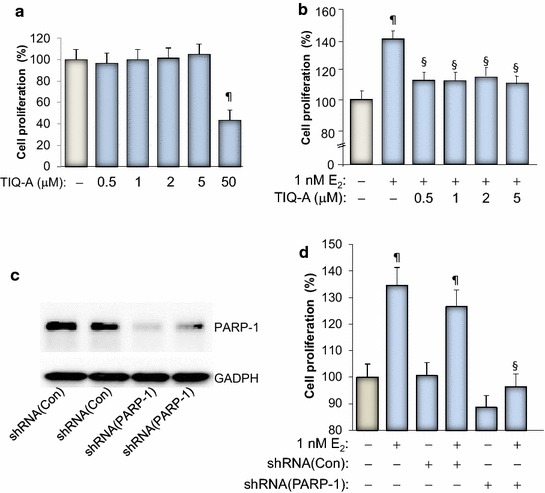
Effect of PARP inhibition on E_2_-stimulated growth of MCF-7 cells. **a** MCF-7 cells were treated for 48 h in the absence or presence of increasing concentrations of TIQ-A. Cell viability was assessed using a MTT assay. ^¶^Difference from viability values of cells that did not receive TIQ-A; *p* < 0.05. **b** MCF-7 cells were stimulated with 1 nM E_2_ for 48 h in the absence or presence of the indicated TIQ-A concentrations. Cell viability was then assessed using a MTT assay. ^¶^Difference from viability values of cells that did not receive TIQ-A; *p* < 0.05. ^§^Difference from viability values of cells that were treated with E_2_ alone; *p* < 0.05. **c** MCF-7 cells were transduced with a viral vector encoding control shRNA or a shRNA targeting human PARP-1. Protein extracts were prepared and subjected to immunoblot analysis with antibodies against PARP-1 or GAPDH. **d** Cells expressing control or PARP-1-targeting shRNA were treated with E_2_ for 48 h after which viability was assessed as described above. ^¶^Difference from respective untreated controls; ^§^ difference from E_2_-treated cells; *p* < 0.05.

Figure 1a shows that PARP inhibition with the specific inhibitor TIQ-A exerted no effect on the growth of the breast cancer cell line MCF-7 except at a very high concentration of 50 μM. This result is consistent with our previous report which concluded that the growth inhibition caused by the high concentrations of PARP inhibitors is most likely associated with toxicity of the drugs rather than an effect related to PARP inhibition [16, 17]. However, it is now established that PARP inhibition causes cell death when combined with BRCA deficiency [18]. It is rather surprising that the role of PARP in estrogen-mediated growth of breast cancer cells has not been investigated. We thus wished to examine whether PARP-1 plays a role in growth mediated by E_2_. Figure 1b shows that E_2_ treatment, as expected, induced ~40% increase in growth of MCF-7 cells, which is typical as reported by our laboratory and others [3, 19]. PARP inhibition by TIQ-A blocked E_2_-induced growth of MCF-7 cells even at a concentration as low as 0.5 μM. The specificity of this effect was confirmed using a PARP-1 shRNA-mediated knockdown approach (Figure 1c, d). These results clearly show that PARP plays a role in E_2_-mediated cell growth and that its inhibition can be regarded as a means to block abnormal proliferation of ER positive breast cancer cells. Such inhibition was achieved with rather low concentrations of the PARP inhibitor. Our results show that in addition to the ability of PARP inhibitor to promote a synthetic lethality phenotype in BRCA-deficient cells [18], it can also reduce E_2_-dependent growth in ER positive tumor cells.

### PARP inhibition-associated reduction in E_2_-induced growth of MCF-7 cells is linked to a reduction in PDZK1 expression with a concomitant decrease in cyclin D1

**Figure 2:**
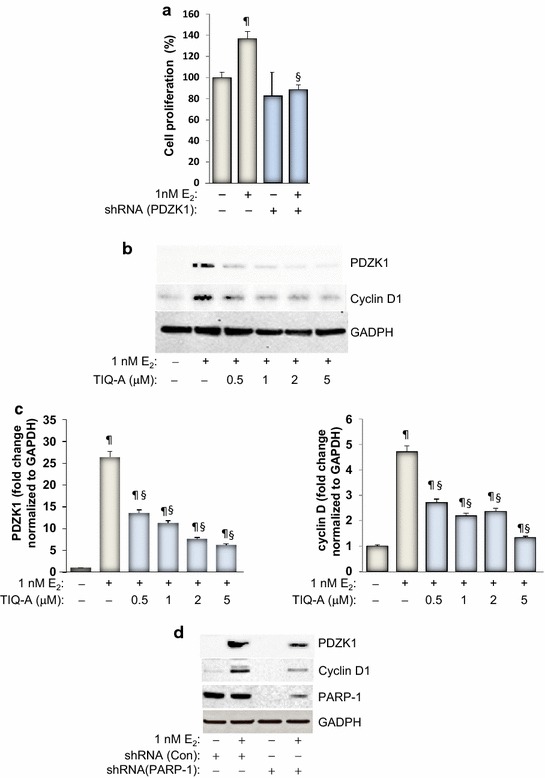
Effect of PARP inhibition on E_2_-stimulated expression of PDZK1 and cyclin D1. **a** MCF-7 cells were transduced with a viral vector encoding control shRNA or a shRNA targeting human PDZK1. Cells were then treated with E_2_ for 48 h after which cell viability was assessed. ^¶^Difference from untreated controls; ^§^difference from E_2_-treated cells; *p* < 0.05. **b** Cells were treated with E_2_ for 48 h after which protein extracts were prepared and subjected to immunoblot analysis with antibodies to PDZK1, cyclin D1, PARP-1, or GAPDH. **c** Cells were treated with E_2_ for 48 h in the absence or presence of increasing concentrations of TIQ-A. Protein extracts were prepared and subjected to immunoblot analysis with antibodies to PDZK1, cyclin D1, or GAPDH. **d** MCF-7 cells were transduced with a viral vector encoding control shRNA or a shRNA targeting human PARP-1. Cells were then treated with E_2_ for 48 h after which protein extracts were prepared and subjected to immunoblot analysis with antibodies to PDZK1, cyclin D1, PARP-1, or GAPDH.

We recently showed in two detailed studies that E_2_-mediated cell growth can be governed by PDZK1 [4]. We thus speculated that PARP-1 may be involved in ER-mediated growth of MCF-7 cells potentially through a control of PDZK1 expression. As reported previously [3] and as shown in Figure 2a, knockdown of PDZK1 blocks growth of MCF-7 cells in response to stimulation with 1 nM of E_2_. We next wished to examine whether PARP-1 enzymatic activity or protein expression influences E_2_-induced PDZK1 expression. To this end, MCF-7 cells were exposed to E_2_ for 48 h in the absence or presence of increasing concentrations of TIQ-A. Immunoblot analysis of collected cell extracts showed that PARP inhibition was very efficient in reducing expression of PDZK1 (Figure 2b, c). The results also show that partial reduction of PDZK1 by 0.5 μM TIQ-A was sufficient to completely block E_2_-induced growth of MCF-7 cells (as shown in Figure 1b). These results are consistent with our previous observation that partial knockdown of E_2_-induced PDZK1 was sufficient to completely block E_2_-induced growth of MCF-7 cells [3]. The involvement of PDZK1 in growth of MCF-7 cells appears to be linked to expression of cyclin D1 as knockdown of PDZK1 prevents E_2_-induced expression of the cell cycle protein [3, 4]. Although it is unlikely that PARP is always required for the expression of cyclin D1, it was recently reported that the expression of the enzyme as well as its activity are required for cyclin D1 expression in response to ectopically-expressed Krüppel-like factor 8 (KLF8) [20]. An additional link between PARP-1 and cyclin D1 is the relationship of both proteins to NF-κB. We and others have shown that PARP-1 plays an important role in NF-κB function [21, 22]. Furthermore, cyclin D1 expression can be controlled by NF-κB [23]. We next examined whether the role of PARP-1 in PDZK1 expression influences expression of cyclin D1 upon E_2_ exposure. Figure 2d shows that PARP inhibition with TIQ-A reduced cyclin D1 expression in a manner similar to that exerted on PDZK1. Additionally, knockdown of PARP-1 markedly reduced expression of PDZK1 and cyclin D1 showing the specificity of the effect (Figure 2d). It is noteworthy that an increase in PDZK1 expression in response to E_2_ treatment did not change the levels of PARP-1 (Figure 2d). This is consistent with our published results showing that ectopic PDZK1 expression in the absence of E_2_ exposure does not affect expression of PARP-1 [4]. Although these results do not prove that E_2_ exposure does not affect the activity of PARP, we can, however, conclude that it does not change the expression levels of this DNA repair enzyme.

### PARP regulates E_2_-induced PDZK1 at the mRNA level

**Figure 3:**
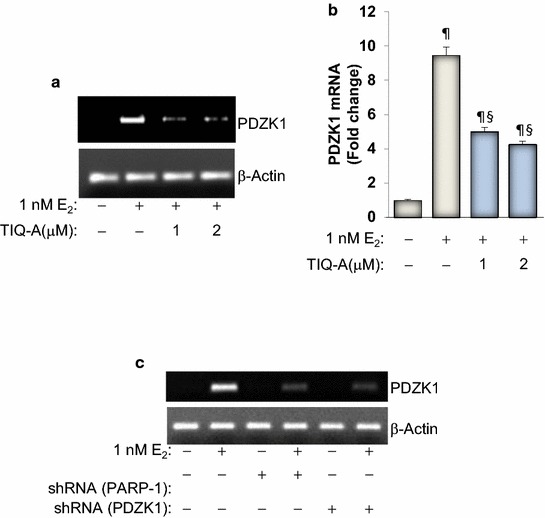
Effect of PARP inhibition on PDZK1 mRNA in E_2_-stimulated MCF-7 cells. MCF-7 cells were treated with E_2_ for 24 h in the absence or presence of 1 or 2 μM TIQ-A. Total RNA was prepared and subjected to reverse transcription followed by conventional (**a**) or quantitative (**b**) PCR with primers specific to human PDZK1 or β-actin. ^¶^Difference from untreated controls; ^§^difference from E_2_-treated cells; *p* < 0.05. **c** MCF-7 cells were transduced with a viral vector encoding control shRNA or a shRNA targeting human PARP-1. Cells were then treated with E_2_ for 24 h after which total RNA was prepared and subjected to conventional RT-PCR with primers specific to human PDZK1 or β-actin.

We have shown previously that PARP-1 may control expression of a number of proteins by regulating their expression at the mRNA level or by influencing their stability. For instance, PARP-1 regulates expression of several inflammatory proteins including adhesion molecules, iNOS, TNF-α, IL-1β, IL-5, and IL-13 [12, 24, 25]. PARP-1 may also regulate proteins at the level of their integrity. Indeed, PARP-1 influences the fate of STAT-6 upon IL-4 or allergen exposure by preventing the degradation of the transcription factor by calpains [26]. We therefore wished to determine whether PARP-1 regulates expression of PDZK1 at the level of mRNA. Figure 3a shows that PARP inhibition was very effective in blocking E_2_-mediated increase in PDZK1 transcript. The effect of the PARP inhibitor on expression of E_2_-induced PDZK1 mRNA was confirmed by quantitative RT-PCR (Figure 3b). Consistent with these results, PARP-1 knockdown was equally efficient in blocking E_2_-mediated increase in PDZK1 mRNA (Figure 3c). These results suggest that PARP-1 may regulate PDZK1 expression at the level of mRNA. However, it is premature to conclude that it influences its gene transcription. Given the lack of evidence for the control of PDZK1 by proteolysis, it is unlikely that PARP-1 regulates the protein by influencing its integrity.

### PARP-1 regulates E_2_-mediated growth of MCF-7 cells and PDZK1 expression by controlling the expression of IGF-1R

**Figure 4:**
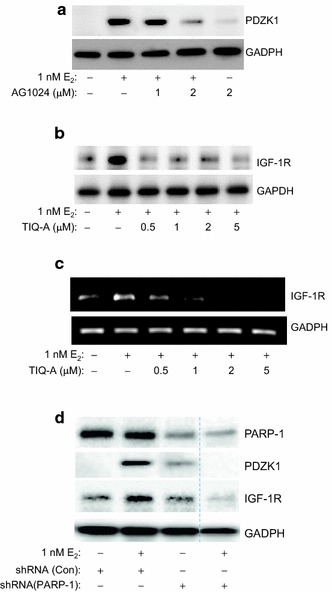
PARP inhibition reduces E_2_-induced IGF-1R. **a** MCF-7 cells were treated with E_2_ for 48 h in the absence or presence of 1 or 2 μM of the IGF-1R inhibitor AG1024 after which protein extracts were prepared and subjected to immunoblot analysis with antibodies to PDZK1 or GAPDH. **b** Cells were treated with E_2_ for 48 h in the absence or presence of increasing concentrations of TIQ-A. Protein extracts were prepared and subjected to immunoblot analysis with antibodies to IGF-1R or GAPDH. **c** MCF-7 cells were treated with E_2_ for 24 h in the absence or presence of 0.5, 1, 2 or 5 μM TIQ-A. Total RNA was prepared and subjected to reverse transcription followed by conventional PCR with primers specific to human IGF-1R or β-actin. **d** MCF-7 cells were transduced with a viral vector encoding control shRNA or a shRNA targeting human PARP-1. Cells were then treated with E_2_ for 48 h after which protein extracts were prepared and subjected to immunoblot analysis with antibodies to PARP-1, PDZK1, IGF-1R, or GAPDH. The *dashed line* indicates that the lane was cut from the same blot.

We reported that PDZK1 expression was indirectly regulated by ER-α activation, requiring IGF-1R expression and function [3]. Blocking the activity of IGF-1R by the specific inhibitor AG1024 blocked E_2_-induced PDZK1 in MCF-7 cells (Figure 4a). We thus speculated that PARP-1 may be regulating PDZK1 expression through IGF-1R. Indeed, PARP-1 inhibition with TIQ-A reduced expression of E_2_-induced IGF-1R even at the lowest concentration (Figure 4b). Figure 4c shows that PARP inhibition efficiently blocked E_2_-induced expression of IGF-1R mRNA in MCF-7 cells. PARP-1 knockdown almost completely blocked the expression of E_2_-induced IGF-1R (Figure 4d) confirming the results attained using the PARP inhibitor and demonstrating specificity of the effect.

### PARP inhibition by olaparib (AZD2281) blocks E_2_-mediated growth of MCF-7 and BT474 cells and inhibits expression of PDZK1 and IGF-1R

**Figure 5:**
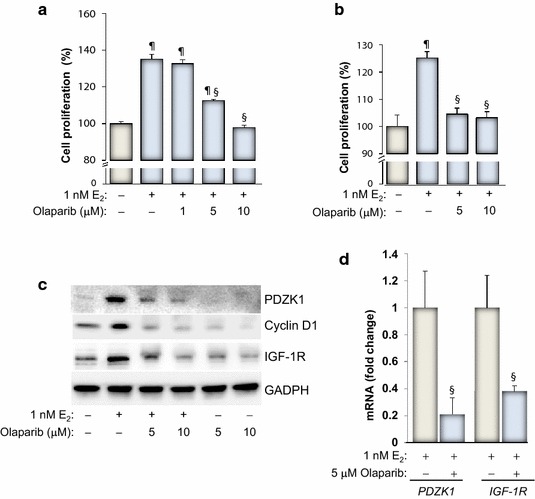
Olaparib treatment blocks E_2_-mediated growth of MCF-7 and BT474 cells and inhibits expression of PDZK1, IGF-1R, and cyclin D1. MCF-7 (**a**) or BT474 (**b**) cells were stimulated with 1 nM E_2_ for 48 h in the absence or presence of the indicated olaparib concentrations. Cell viability was then assessed using a MTT assay. ^¶^Difference from viability values of cells that did not receive olaparib; *p* < 0.05. ^§^Difference from viability values of cells that were treated with E_2_ alone; *p* < 0.05. **c** MCF-7 cells were treated with E_2_ for 48 h in the absence or presence of increasing concentrations of olaparib after which protein extracts were prepared and subjected to immunoblot analysis with antibodies to PDZK1, cyclin D1, IGF-1R, or GAPDH. **d** MCF-7 cells were treated with E_2_ for 24 h in the presence of 5 μM olaparib. Total RNA was prepared and subjected to reverse transcription followed by quantitative PCR with primers specific to human PDZK1, IGF-1R or β-actin. ^§^Difference from E_2_-treated cells; *p* < 0.05.

An important step in proving the validity of targeting PARP to control growth of ER(+) breast cancer cells is to demonstrate that PARP inhibitors other than TIQ-A can achieve the same effect and to show that such effect is observed in breast cancer cells other than MCF-7 cells. Olaparib (AZD2281), a potent PARP inhibitor, is currently used in a large number of clinical trials targeting BRCA(−) breast and ovarian cancers [15]. We thus tested the effect of different concentrations of olaparib on growth of MCF-7 cells. Figure 5a shows that olaparib at the 5 μM concentration partially but significantly reduced E_2_-induced cell growth; the 10 μM concentration of the drug completely blocked growth of the treated cells. Figure 5b shows that olaparib was very effective in blocking growth of BT474 cells, another ER(+) cell line [27].

We next examined whether growth inhibition of MCF-7 cells by olaparib correlated with a decrease in PDZK1, cyclin D1 and IGF-1R. Figure 5c clearly shows that olaparib treatment markedly blocked expression of E_2_-induced PDZK1 in a manner similar to that achieved by TIQ-A. Such inhibition of PDZK1 expression occurred concomitantly with a reduction in cyclin D1 and IGF-1R upon exposure to E_2_ in MCF-7 cells. Figure 5d shows that PARP inhibition reduces PDZK1 and IGF-1R at the level of mRNA. Overall, these results are consistent with those attained using TIQ-A as a PARP inhibitor and demonstrate that the ability of PARP inhibition to block E_2_-induced growth is not limited to TIQ-A or MCF-7 cells but may be extended to other ER(+) breast cancer cells.

## Conclusions

It is well recognized that breast and ovarian cancer heterogeneity continues to represent a major obstacle in tailoring precise and efficient therapies with minimal toxicities. Much effort is needed to identify key determining factors that control the growth of cancer cells. Accordingly, insights on the role of new factors or identification of new functions for already established players may provide us with new directions and strategies with which cancer can be retarded or blocked. Collectively, our results suggest that PARP may play an important role in E_2_-induced cell growth by regulating the expression of PDZK1 through a control of the stimulated expression of IGF-1R. To our knowledge, our results are the first in showing a connection between PARP and ER-stimulated growth and that such trait may be considered in the current effort in establishing PARP inhibitors in the treatment of breast and ovarian cancers. We have shown a strong relationship between PDZK1 and IGF-1R expression in human breast cancer and that PDZK1 may be a determinant in breast tumorigenesis [3, 4]. The molecular link between PDZK1 and IGF-1R was supported by a significant correlation between protein and mRNA levels of the two factors in two independent cohorts of human breast cancer tissues [3].

The current results suggest that the relationship between ER, PDZK1, and IGF-1R may be an appealing axis to be targeted by PARP inhibitors. It is noteworthy that a great deal of effort is being spent focusing primarily on triple negative breast and ovarian cancers with a BRCA deficiency to specifically achieve synthetic lethality of cancer cells with the BRCA mutation [28]. Although the presented results do not provide a complete understanding of the mechanism by which PARP regulates PDZK1 or IGF-1R, they support the hypothesis that PARP inhibitors, including olaparib, may be useful for treatment of ER(+) and estrogen-dependent cancers. Obviously, more work is needed to validate such hypothesis and unravel the molecular mechanisms underlying the cross-talk between the different factors.

## Abbreviations

PARP: poly(ADP-ribose) polymerase
ER: estrogen receptor
IGF-1R: IGF-1 receptor
TIQ-A: thieno[2,3-c]isoquinolin-5-one
